# PAs as principal investigators of FDA-regulated clinical trials

**DOI:** 10.1097/01.JAA.0000931416.49268.b2

**Published:** 2023-05-25

**Authors:** Colleen Elizabeth Gosa Nalepinski

**Affiliations:** **Colleen Elizabeth Gosa Nalepinski** is decentralized clinical trials director in research nursing and phlebotomy solutions at IQVIA in Durham, N.C.; a clinical assistant professor at Marquette University in Milwaukee, Wisc.; and a principal investigator at Spaulding Clinical Research in West Bend, Wisc. The author has disclosed no potential conflicts of interest, financial or otherwise.

**Keywords:** physician associate, physician assistant, principal investigator, clinical trial, FDA, research

## Abstract

A misconception exists that only physicians can be principal investigators for FDA-regulated human clinical trials such as interventional studies. This article reviews existing guidelines and dispels the notion that physician associates/assistants (PAs) cannot be principal investigators for clinical trials. Additionally, this article describes an implementation plan to correct the misconception and establish a reference for future PAs seeking the role of principal investigator in clinical trials.

The FDA states that a principal investigator for a human clinical trial is required to be qualified by training, education, and experience but does not define constraints for these three areas.^1^ Per the FDA, the study sponsor determines if a principal investigator candidate qualifies for clinical trials on investigational new drugs or biologic agents. Historically, physician associates/assistants (PAs) have served as subinvestigators in clinical trials, gaining training and experience that can prepare them for serving as principal investigators. However, uncertainty persists both industrywide and within the PA profession, about whether PAs can be principal investigators of clinical trials. The lack of clarity is a deterrent for PAs and institutions. Regulations do, in fact, permit PAs to be principal investigators of clinical trials.

## LITERATURE AND DATABASE SEARCH

A literature search of MEDLINE, PubMed Central, and WorldCat.org using *principal investigator/PI* and *physician assistant/PA* as keywords and title yielded zero relevant articles. The search was limited to peer-reviewed journals published in English. The clinicaltrials.gov website was searched for PAs listed as principal investigators, and found eight PAs listed as principal investigators.^2^ At the time of this search, the database of 145,398 phase 1 through 4 interventional studies for “PA-C” yielded 14 instances of a principal investigator with PA-C credentials. The 14 trials were done by eight PAs, with start dates between 2007 and 2020 with stop dates between 2008 and 2023. Of the eight PA principal investigators, four handled postmarketing studies. Funding was listed as *other* for 10 of the 14 studies, *US federal* for 2, and *other/industry* for 2. None of the PAs listed doctorate credentials. A notable limitation of this search is that not all clinical trials are registered. Additionally, the National Institutes of Health does not keep a record of investigator's credentials, and *investigator* is not a search field.

The search further revealed that no PAs have served as principal investigator for solely industry-funded phase 1 through 4 clinical trials on an FDA-regulated drug, biologic, or medical device. Guidance permits PAs to serve as principal investigators, but the industry may lack awareness or have internal standard operating procedures that limit the role to physicians. The industry's internal procedures also may be a barrier.

Observational studies and other academic research on approved drugs and biologics do not require an FDA Form 1572 and sponsor principal investigator approval. The scope of this article excludes observational studies and behavioral and device trials indicated as phase *not applicable* on clincialtrials.gov.

Over the past few decades, there has been an increase in PAs who are principal investigators for observational research studies. PA principal investigators in academia are a laudable example of the expanding role of the PA profession. This article focuses on human clinical trials conducted in phases 1 through 4.

## RESPONSIBILITIES, SELECTION, AND APPROVAL

The principal investigator is responsible for conducting a clinical trial and agrees to complete the trial following FDA regulations and good clinical practices. The signed FDA Form 1572 is the principal investigator's contractual record. The FDA expects the principal investigator to “supervise a clinical study in which some study tasks are delegated to employees or colleagues of the investigator or other third parties and ... to protect the rights, safety, and welfare of study subjects.”^1^ The safety decisions can be led and supported by a study physician who can collaborate with a team of clinicians.

The principal investigator is designated by completion of two steps—selection by the sponsor and approval by the institutional review board (IRB). The International Council for Harmonization (ICH) E6 guideline states that a physician must make medical decisions for the trial; however, it does not state that the principal investigator must be a physician.^3^ The E6 guideline also states that the sponsor is responsible for selecting the investigator(s) and institutions. The FDA notes that each investigator is qualified by training and expertise. Additionally, the principal investigator should have adequate resources to conduct the trial.^3^

The IRB is responsible for reviewing the principal investigator's qualifications as part of the trial application to the IRB. To determine if the principal investigator is qualified, the IRB can ask questions and request documents such as a curriculum vitae.^4^

## GUIDANCE

The FDA and ICH are the keystones of good clinical practices in the United States. Relevant clinical trial and investigator guidance for prohibitive language were reviewed, as well as the principal investigator scope and required qualifications. The purpose of the study physician role was detailed to meet the FDA requirement for trials with a nonphysician principal investigator. Lastly, a comparable healthcare profession inquiry was reviewed.

FDA guidance requires a principal investigator to be qualified by training, education, and experience.^1^ As stated earlier, the FDA does not explicitly define qualifications for these three categories. The study sponsor determines principal investigator qualification by reviewing the investigator's curriculum vitae. No FDA guidance documents state education requirements; for example, a medical degree is not required. In response to a query, a senior health policy analyst at the FDA referenced the guidance document, which states, “The regulations do not require that the investigator be a physician.”^5^

A frequent assumption is that a principal investigator's education consists of attending medical school. *Principal investigator* and *physician* are used synonymously in literature and conversation. Journal database searches yield key examples of ICH E6 incorrectly referenced by paraphrasing the document.

FDA regulations are considered the minimum standards for conducting clinical research. PA education is based on the medical model, and PAs are licensed to diagnose, develop a treatment plan, and prescribe. PA certification demonstrates sufficient evidence to be qualified by education to be a principal investigator.

The ICH was held in 1990 and has had several updates since. The purpose of the inaugural conference was “to protect the rights of human subjects participating in clinical trials and ensure the scientific validity and credibility of the data collected in human clinical studies.”^6^ ICH guidance defines good clinical practices, stating that medical decisions are made by a qualified physician. The FDA developed the E6 guidance document within the ICH's Expert Working Group (Procedural), and the ICH has endorsed it. E6 reiterates that a qualified physician must make medical decisions. The study physician role satisfies this requirement, and subsequently, the principal investigator does not also have to be a physician. The FDA Guidance for Industry E6 Good Clinical Practice states that a study physician should be a subinvestigator when the principal investigator is not a physician. The physician is responsible for medical decisions in the trial.^3^

Interestingly, pharmacists made a similar inquiry to the FDA in 1983, asking if they could be principal investigators of clinical trials. The FDA responded that “it has long been FDA policy to accept doctors of pharmacy as primary investigators of studies of investigational drugs within their area of expertise.”^7^ However, local regulations (state, local, health system, or IRB policies and procedures) can prevent nonphysicians, such as pharmacists, from being principal investigators.^8^ The *American Journal of Hospital Pharmacy*, in 1989, published the successful petitioning of a state board to align with the FDA guidance.^9^ Similarly, PAs should verify state regulations and petition when necessary.

## IMPLEMENTATION

PAs pursuing the principal investigator role should gain clinical trial experience in the subinvestigator role and complete good clinical practices training. As subinvestigators, PAs can gain experience and knowledge by working closely with trial staff. This exposure will help them learn the research specialty. Participating in weekly sponsor teleconferences, site initiation visits, investigator meetings, and operational discussions help PAs gain experience. Additional training and certifications, such as certified principal investigator, can be helpful but are not required. For PAs working at institutions where the principal investigator role is synonymous with the physician role, the recommended implementation steps are:

- Request a revision to the principal investigator job description to broaden degree requirements.
- Review principal investigator work instructions and standard operating procedures for necessary document change requests.
- Create a study physician job description.
- Develop and revise a principal investigator training plan (this may include moving physician training items to a study physician training plan).
- On the delegation of authority log, create the study physician role. Reference appliable guidance and publications in change requests.

## CONCLUSION

A review of relevant guidance determined no prohibitive language preventing PAs from being principal investigators of FDA-regulated human clinical trials. FDA regulations do not require the principal investigator to be a physician. The degree a candidate holds is not the determining factor. Training, education, and experience qualify a principal investigator candidate. Both FDA and ICH guidance anticipated nonphysician scenarios and thereby stated the requirement of the study physician role. PAs, albeit few, have been principal investigators of clinical trials. These findings support that a PA can be a principal investigator of interventional studies.

PA practice has evolved significantly over the years. Many PAs working on clinical trials desire to expand their role. Additionally, more principal investigators are needed, and PAs can meet this need. More importantly, PAs are an appropriate fit for satisfying the regulatory requirements and associated responsibilities. Improved awareness is essential for more PAs to step in as principal investigators in clinical trials. PAs, as prospective principal investigators, can reference guidance to correct misconceptions, implement changes, and expand their role in clinical trials.
